# Using individualised bowel care plans to improve clinical outcomes in specialist intellectual disability mental health units in England and Wales: quality improvement project

**DOI:** 10.1192/bjo.2025.10814

**Published:** 2025-08-18

**Authors:** Alexandra Gabrielsson, Richard Laugharne, Jon Painter, Harriet Slater, Catherine Bright, Andrew Dossett, Romanie Dekker, Alex Bordessa-Kelly, Kloe Edwards, Jarod Newbury, Salman Azfar, Paul Bassett, Samuel Tromans, Indermeet Sawhney, Phil Elliot, Mahesh Odiyoor, Kiran Purandare, Rohit Shankar

**Affiliations:** Hertfordshire Partnership University NHS Trust, Hatfield, UK; Cornwall Partnership NHS Foundation Trust, Truro, UK; Cornwall Intellectual Disability Equitable Research (CIDER), University of Plymouth, Truro, UK; School of Health and Social Care, Sheffield Hallam University, Sheffield, UK; Aneurin Bevan University Health Board, Newport, UK; Sussex Partnership NHS Foundation Trust, Chichester, UK; Mildmay Oaks, Hook, UK; Stats Consultancy, Buckinghamshire, UK; University of Leicester, Leicester, UK; Leicestershire Partnership NHS Trust, Leicester, UK; Cheshire and Wirral Partnership NHS Foundation Trust, Chester, UK; Central and Northwest London Foundation NHS Trust, London, UK

**Keywords:** Premature mortality, in-patient, challenging behaviour, neurodevelopmental disorders, quality of life

## Abstract

**Background:**

Constipation is a significant problem for people with intellectual disabilities, with a prevalence of 33–50%, causing at least five deaths annually in England. Individualised bowel care plans (IBCP) are recommended in England and Wales.

**Aims:**

We evaluated the feasibility and impact of IBCPs for people with intellectual disabilities who are in in-patient psychiatric units, and the effect on clinical outcomes.

**Method:**

People with intellectual disabilities who were at risk of constipation were recruited from four specialist in-patient psychiatric units in England and Wales. A constipation questionnaire was used to capture relevant data to devise IBCPs. Baseline, 3- and 6-monthly Health of the Nation Scales – Learning Disability (HoNOS-LD) were completed after the intervention. Descriptive statistics, Wilcoxon signed-rank, Mann-Whitney *U*, repeated-measures analyses of variance, with Bonferroni adjustment and Mauchly’s tests were conducted. Significance was taken at *P* < 0.05.

**Results:**

Of 24 people with intellectual disabilities recruited from four units, all three data points were available for 18 patients. Constipation rates showed no statistically significant decline. The total HoNOS-LD score (18 items) did not decline. HoNOS-LD item 12 for physical functioning showed significant improvement for PwID with constipation compared with those without, between baseline and 6 months.

**Conclusions:**

This quality improvement project suggests that a bigger study of IBCPs is feasible. Most outcomes examined via the HoNOS-LD, particularly those linked with mental illness, challenging behaviour and quality of life, did not show significant change, possibly because of the small sample size. However, people with intellectual disabilities and constipation showed positive changes in their physical functioning outcomes compared with those without constipation. Further in-depth evaluation of this intervention is needed.

Intellectual disability is defined as a neurodevelopmental condition, originating in childhood, which leads to deficits in intellectual, social and adaptive functioning,^1^ affecting the individual across all aspects of life. People with intellectual disabilities (PwID) are a heterogenous population, and the impact of their intellectual disability on each of them varies widely, from those requiring minimal support in daily life to those who need continuous care and support. Despite the heterogeneity within the group, many individuals across the spectrum of intellectual disability share the challenges of accessing equitable health services in their communities.^2^ The 2022 Learning from Lives and Deaths – People with a Learning Disability and Autistic People (LeDeR) report into avoidable deaths of people with intellectual disabilities estimated that 42% of deaths of PwID were avoidable, compared with 22% in the general population.^3^ These are concerning statistics for a population in which co-occurring and complex mental and physical health problems commonly occur.^4,5^

## Constipation and intellectual disability

Constipation is a heterogenous condition characterised by uncomfortable and/or infrequent bowel movements.^6^ PwID are more prone to constipation than the general population.^7^ Prevalence estimates of 33–50% are reported in the literature, and rise further in people with severe and profound intellectual disability.^8,9^ Concerningly, constipation is also one of the top five causes of hospital admission in PwID,^10^ and can have life-threatening consequences if left untreated.^11^ The LeDeR review found that 23% of deaths in PwID identified constipation as a long-term problem, and 33% were taking laxatives at the time of death.^12^ The 2019 LeDeR report^13^ recorded 12 deaths directly caused by constipation over a 5-year period; a worrying mortality figure for a condition that is both avoidable and curable.

Like other chronic health condition management, constipation is contingent on prevention, or where this is not possible, early identification of possible risk factors and suitable treatment.^14,15^ Proactive prevention and management of constipation has been suggested to be an effective way of improving quality of life for PwID.^16^ The current evidence base on the management of constipation in PwID is sparse, and the mainstay of constipation management in this cohort continues to be pharmacological interventions, despite a lack of robust evidence supporting their effectiveness in this group of patients^8^ and concerns around unsystematic approaches to laxative prescribing in PwID.^17^

NHS England has developed materials specifically for PwID and their family/carers, aimed at identifying the signs of constipation and treating the condition proactively.^11^ They recommend that all PwID who are at risk of constipation have an individualised bowel care and escalation plan (IBCP)^18^ to facilitate a holistic treatment approach to constipation.

Admission to a specialist psychiatric in-patient mental health units for PwID (defined for the purpose of this project as ‘in-patient units’) represents a time of enhanced behavioural/psychiatric distress, associated with considerable psychological, environmental, lifestyle and pharmacological changes.^19^ These are all factors which have the potential to adversely affect bowel health. The opportunity for close clinical monitoring and availability of clinical expertise is relatively unique to the in-patient setting, and provides a good opportunity for the implementation of IBCPs and to explore their potential to improve bowel health in PwID.

We sought to evaluate the feasibility and effectiveness of implementing IBCP on constipation and clinical outcomes for PwID in in-patient settings. We chose a feasibility-based method as an established way of exploring the appropriateness, relevance and sustainability of an intervention.^20^ We also examined if IBCP changes constipation status, and is associated with improved clinical outcomes at 3 and 6 months after their introduction.

## Method

The SQUIRE 2 (Standards for QUality Improvement REporting) guidelines, a framework for reporting on studies that improve healthcare quality (Supplementary File 1 available at https://doi.org/10.1192/bjo.2025.10814), was used to design and execute this study.^21^

### Recruitment and participants

In-patients on participating units were invited to complete the questionnaire, with support from carers and staff as needed. Patients who were coming to the end of their admission were not included, to avoid loss to follow-up at 3 months.

Informed consent was sought from prospective participants, using easy-to-read material and infographics created specifically for the purpose of the project. The material was introduced to the patient by a member of the multidisciplinary team (nurse, doctor or occupational therapist) who was familiar with the patient. For participants who did not have capacity to consent to the project, assent was sought from family members and the full multidisciplinary team through the best interest decision process. For consenting participants, their family were also informed of the project and were provided with relevant information leaflets. Although this project was not classed as research and no formal informed consent was needed, the investigating team agreed that attempts to inform participants of the study was an important part of the project process because it empowers patient’s decision-making, respects their autonomy and can help improve engagement with treatment plans in the evaluation.^22^ Eligibility of a patient included a past or current history of chronic constipation. The decision on eligibility was taken by each participating site investigator based on the medical records available to them.

### Data collection tools

Constipation was defined as having fewer than three bowel movements in a week and/or taking laxatives three or more times per week. The Constipation Questionnaire, a purpose-designed questionnaire covering a range of biopsychosocial factors relevant to constipation as well as a measure of constipation, created collaboratively by a multidisciplinary expert consensus group, was used.^23^ The questionnaire is provided in Supplementary File 2. From this questionnaire, an IBCP was completed by staff based on the NHS England template circulated by the project team (^18^, Supplementary File 3). An IBCP is a holistic, personalised way of managing a person’s bowel health by taking factors such as diet, toileting routine, exercise and medication into account. The plan highlights risk factors for constipation in the individual and outlines a plan of action should the person become constipated. Where possible, the plans were co-produced by the patient and members of the multidisciplinary team. This was implemented across the 6 months of the project.

The Health of the Nation Scales – Learning Disability (HoNOS-LD),^24^ a clinician-rated clinical outcome tool, was used to measure change over the project period. The HoNOS-LD is a clinician-reported outcome measure, which has been found to be a valid and reliable measure of health and social functioning for PwID, including if they have co-occurring autism or additional mental health needs.^25,26^

Data were collected at baseline, and after 3 and 6 months following implementation of the IBCPs, to explore effects on constipation status and HoNOS-LD scores.

### Data analysis

The analyses focused on the change in outcomes from baseline to both the 3- and 6-month time points of the constipation questionnaire data and the HoNOS-LD.

### Constipation questionnaire analysis

Continuous variables are summarised by the mean and standard deviation, and the number and percentage in each category are reported for the categorical variables. Binary categorical outcomes were compared between time points by using the paired exact test, because of the relatively small numbers in some categories. Other outcomes were ordinal in nature, and these were compared between time points by using the Wilcoxon signed-rank test. Significance was taken at *P* < 0.05.

The recorded medication for each participant has been collated into Supplementary File 4. Anticholinergic burden (ACB) scores have been calculated for each participant at each data collection point, using the ACB Calculator. The ACB Calculator, which can be readily accessed online, is a validated tool used to assess the risk of cumulative adverse effects from medication with anticholinergic effects, which can contribute to cognitive impairment and overall mortality.^27^

### HoNOS-LD analysis

For each participant, total scores were calculated for their baseline, mid-point and end-point HoNOS-LD ratings to quantify their overall symptom severity at each point. Repeated-measures analyses of variance (ANOVA), with Bonferroni adjustment, were then used to identify whether the changes in HoNOS-LD totals across the three time points were statistically significant. These tests were performed on the full data-set, the subset of participants with constipation at baseline and the subset without constipation at baseline. Using the overall symptom severity variables (described above), additional ‘change variables’ were calculated for the change in HoNOS-LD totals between baseline and mid-point, as well as from baseline to end-point. These two ‘change variables’ were then analysed with Mann-Whitney *U*-tests to see whether the level of change (independent of symptom severity) differed significantly between participants with and without constipation at baseline.

Finally, this two-part analysis was repeated solely using item 12 of the HoNOS-LD rating scale, recorded at each time point, as this ‘Physical Problems’ scale is where constipation should be rated. Mauchly’s test of sphericity was used to test if the assumption of sphericity was met in a repeated-measures ANOVA. Significance was taken at *P* < 0.05.

### Ethics and governance

This study did not require formal ethical approval as per the NHS Health Research Authority tool (Supplementary File 5). One site Principal Investigator first registered it formally as service evaluation/quality improvement in their NHS Trust (Supplementary File 6). Following this, members of the project team with affiliations to one or more intellectual disability in-patient units then registered the approved protocol and associated documentation as a service improvement project in their respective NHS Trusts and health organisations. Each participating centre included in the study registered conducted a Data Protection Impact Assessment and/or any other needed local governance assessments. Data were anonymised at source. Only de-identified data were submitted to the database. Data were collected in compliance with the General Data Protection Regulation.

The authors assert that all procedures contributing to this work comply with the ethical standards of the relevant national and institutional committees on human experimentation and with the Helsinki Declaration of 1975, as revised in 2013.

## Results

### Baseline findings

Twenty-four patients were recruited from four hospitals, three in England and one in Wales, between January and December 2024. The total patient capacity of the four sites is 72 beds. The hospital-specific bed availability, recruitment and drop-out data are provided in Table 1.

**Table 1:** Details of participating hospitals and recruitment

Site	In-patient beds	Patients recruited	Discharges across 6 months	Other loss to follow up
Sussex	5	5	0	0
Wales	12	12	0	1^a^
Hertfordshire	14	4	1^b^	0
Hampshire	41	3	0	0
Total	72	24	1	1

a .One participant died following baseline collection from causes unrelated to constipation.

b .One was discharged after 3 months and thus lost for the 6-month follow-up.

Demographic information was collected by clinicians familiar with the patient, supplemented by information from the electronic patient records as required. A summary of participant characteristics is presented in Table 2. The participants had a mean age of 41 years, with over two-thirds (71%, *n* = 17)) being male. Half of the participants (*n* = 12) had mild intellectual disability and four had a genetic syndrome including Down syndrome (16%). Three-quarters had a documented mental illness (*n* = 18). Half of patients (*n* = 12) were obese and 29% (*n* = 7) of participants had epilepsy. The constipation questionnaire baseline data, before the introduction of the IBCPs, is provided in Table 3. None of the participants had been admitted to hospital for constipation. Two patients had fewer than three bowel motions in a week and nine took laxatives three or more times weekly. A total of ten patients satisfied the definition of constipation and one patient satisfied the criteria for constipation and use of regular laxatives. Thirteen (59%) participants had faecal incontinence and 12 (52%) used laxatives weekly. The majority of participants (84%, *n* = 20) had received dietary advice and for two-thirds (67%, *n* = 16) of those, the advice had been implemented. Most participants (83%, *n* = 15/18) toileted independently, whereas a minority (17% *n* = 3/18) required support; 17% (*n* = 4/24) were reported to have an assisted toileting routine, and 83% (*n* = 20/24) did not. Most of the respondents (95%, *n* = 18/19) used a normal toilet seat. Nearly a third (29%, *n* = 7/23) had impaired mobility or were largely immobile. A total of 86% (*n* = 18) of participants had an ACB score of 3 or above, which confers an increased risk of overall impairment, falls and mortality.

**Table 2:** Participant demographics

Variable	*n*	Category	Summary mean ± s.d. or *n* (%)
Age	24	–	41.8 ± 14.8
Gender	24	Male	17 (71)
		Female	7 (29)
Degree of intellectual disability	24	Mild	12 (50)
		Moderate	5 (21)
		Severe-profound	7 (29)
Down syndrome	24	No	22 (92)
		Yes	2 (8)
Any other known genetic syndrome	24	No	22 (92)
		Yes	2 (8)
Cerebral palsy	24	No	18 (75)
		Yes	6 (25)
Epilepsy	24	No	17 (71)
		Yes	7 (29)
Mental illness	24	None	6 (25)
		Non-psychotic	13 (54)
		Psychosis	5 (21)
Dysphagia	24	No	19 (79)
		Yes	5 (21)
Obesity	24	No	12 (50)
		Yes	12 (50)
Diabetes	24	No	21 (87)
		Yes	3 (13)
Autism	23	No	11 (48)
		Yes	12 (52)

Those participants who were able and willing to participate were invited to complete the questions collaboratively with multidisciplinary team members whom they worked closely with. For those participants who could not directly contribute because of more severe-profound intellectual disability, information was predominantly completed from the multidisciplinary team’s clinical knowledge of the patient, family members and electronic patient records.

**Table 3:** Constipation questionnaire variables at baseline

Variable	*n*	Category	*n* (%)
Bowel movements per week	21	≤2	2 (10)
		>2	19 (90)
Faecal incontinence	22	Daily	8 (36)
		Less than daily	5 (23)
		Never	9 (41)
Laxative use	23	3+ per week	9 (39)
		<3 per week	3 (13)
		Never	11 (48)
Constipation leading to admission	24	No	24 (100)
		Yes	0 (0)
Diet advice	24	No advice	4 (17)
		Advice, not implemented	4 (17)
		Advice, implemented	16 (67)
Person giving diet advice	20	Balanced diet advice	5 (25)
		Dietician	8 (40)
		Doctor	1 (5)
		Doctor + speech therapist	2 (10)
		SALT	2 (10)
		SALT + dietician	2 (10)
Fluid intake	23	Intake difficult	4 (17)
		Intake good	19 (83)
Toileting: independence	18	Requires support	3 (17)
		Independently toilets	15 (83)
Toileting: routine	24	Assisted routine	4 (17)
		No routine	20 (83)
Toileting: seat	19	Raised seat	1 (5)
		Normal seat	18 (95)
Mobility: exercise	23	Impaired	6 (25)
		Largely immobile	1 (4)
		Good with exercise	6 (25)
		Good without exercise	11 (46)
Residence	24	Hospital	23 (96)
		Supported living	1 (4)

SALT, speech and language therapy.

#### Change from baseline to 3 months

Results of changes in outcome between the baseline time point and 3 months after the IBCPs were introduced are provided in Table 4. None of the outcomes showed a significant difference between baseline and 3 months. Nonetheless, looking at the numbers, there were some positive developments. More patients had diet advice (baseline *n* = 20/24, 3 months *n* = 20/23, 6 months *n* = 16/17) and more had this advice implemented at 3 months, although numbers declined by the 6-month mark (baseline *n* = 16/20, 3 months *n* = 17/20, 6 months *n* = 13/17). More patients had a toilet routine (baseline *n* = 4/24, 3 months *n* = 8/21, 6 months *n* = 12/18) and fluid intake improved at 3-month data collection (baseline *n* = 19/23, 3 months *n* = 21/22, 6 months *n* = 17/18). For 86% (*n* = 18) of participants, the ACB scores remained stable after 3 months: 9.5% (*n* = 2) saw their ACB score increased by 2 and 3 points, respectively, and 5.5.% (*n* = 1) showed a 5-point reduction in ACB score following medication changes.

**Table 4:** Comparison of outcome measures at baseline and 3 months

Variable	*n*	Category	Baseline *n* (%)	3 months *n* (%)	*P*-value
Bowel movements per week	20	≤2	2 (10)	2 (10)	1.00
		>2	18 (90)	18 (90)	
Faecal incontinence	21	Daily	7 (33)	6 (29)	0.50
		Less than daily	5 (24)	4 (19)	
		Never	9 (43)	11 (52)	
Laxative use	22	≥3 per week	9 (41)	9 (41)	0.50
		<3 per week	3 (14)	1 (5)	
		Never	10 (45)	12 (55)	
Constipation leading to admission	23	No	23 (100)	23 (100)	1.00
		Yes	0 (0)	0 (0)	
Diet advice	23	No advice	4 (17)	3 (13)	1.00
		Advice	19 (83)	20 (87)	
Diet advice implemented	23	None/not implemented	8 (35)	6 (26)	0.69
		Implemented	15 (65)	17 (74)	
Fluid intake	22	Intake difficult	3 (14)	1 (5)	0.50
		Intake good	19 (86)	21 (95)	
Toileting: independence	18	Requires support	3 (17)	6 (33)	0.25
		Independent	15 (83)	12 (67)	
Toileting: routine	21	Assisted routine	4 (19)	8 (38)	0.22
		No routine	17 (81)	13 (62)	
Toileting: seat	19	Raised seat	1 (5)	1 (5)	1.00
		Normal seat	18 (95)	18 (95)	
Mobility: exercise	23	Impaired	5 (22)	6 (26)	0.36
		Large immobile	1 (4)	1 (4)	
		Good with exercise	6 (26)	8 (35)	
		Good without exercise	11 (48)	8 (35)	
Residence	23	Hospital	22 (96)	22 (96)	1.00
		Supported living	1 (4)	1 (4)	

#### Change from baseline to 6 months

Outcomes at baseline and 6 months were compared and are provided in Table 5. The results suggested no significant differences between baseline and 6 months for any of the outcomes examined. There were some positive movements as for the 3-month data: the number of patients with good fluid intake improved from 15 out of 18 patients to 17 out of 18 patients.

**Table 5:** Comparison of outcome measures at baseline and 6 months

Variable	*n*	Category	Baseline *n* (%)	6 months *n* (%)	*P*-value
Bowel movements per week	17	≤2	2 (12)	1 (6)	1.00
		>2	15 (88)	16 (94)	
Faecal incontinence	17	Daily	6 (35)	5 (29)	0.75
		Less than daily	4 (24)	4 (24)	
		Never	7 (41)	8 (47)	
Laxative use	13	≥3 per week	2 (15)	2 (15)	0.50
		<3 per week	3 (23)	1 (8)	
		Never	8 (62)	10 (77)	
Constipation leading to admission	18	No	18 (100)	18 (100)	1.00
		Yes	0 (0)	0 (0)	
Diet advice	17	No advice	1 (6)	1 (6)	1.00
		Advice	16 (94)	16 (94)	
Diet advice implemented	17	None/not implemented	5 (29)	4 (24)	1.00
		Implemented	12 (71)	13 (76)	
Fluid intake	18	Intake difficult	3 (17)	1 (6)	0.50
		Intake good	15 (83)	17 (94)	
Toileting: independence	14	Requires support	3 (21)	4 (29)	1.00
		Independent	11 (79)	10 (71)	
Toileting: routine	18	Assisted routine	3 (17)	6 (38)	0.25
		No routine	15 (83)	12 (67)	
Toileting: seat	19	Raised seat	1 (7)	1 (7)	1.00
		Normal seat	14 (93)	14 (93)	
Mobility: exercise	19	Impaired	5 (22)	6 (32)	1.00
		Large immobile	1 (5)	1 (5)	
		Good with exercise	5 (26)	4 (21)	
		Good without exercise	8 (42)	8 (42)	
Residence	22	Hospital	21 (95)	21 (95)	1.00
		Supported living	1 (5)	1 (5)	

### Analysis of HoNOS-LD data

Table 6 shows descriptive statistics for the six severity variables and four change variables that were derived from the 24 patients’ HoNOS-LD ratings. The constipated group were consistently more symptomatic than the norm.

**Table 6:** HoNOS-LD descriptive statistics for severity and change variables

Variable	Full data-set					Constipated at baseline					Not constipated at baseline				
	*n*	Minimum	Maximum	Mean	s.d.	*n*	Minimum	Maximum	Mean	s.d.	*n*	Minimum	Maximum	Mean	s.d.
Baseline scale 12 rating	24	0	4	1.42	1.72	10	0	4	2.30	1.77	14	0	4	0.79	1.42
Mid-point scale 12 rating	21	0	4	1.52	1.57	10	0	4	2.20	1.69	11	0	4	0.91	1.22
End-point scale 12 rating	17	0	4	1.82	1.51	8	0	4	2.38	1.69	9	0	4	1.33	1.23
Baseline HoNOS-LD total score	24	7	39	21.37	7.79	10	17	36	25.50	7.65	14	7	39	19.22	9.16
Mid-point HoNOS-LD total score	21	7	37	20.00	7.46	10	16	34	22.50	5.73	11	7	37	20.44	8.96
End-point HoNOS-LD total score	17	3	46	21.76	9.17	8	18	34	22.50	5.35	9	3	46	21.11	11.92
Baseline to mid-point change in HoNOS-LD scale 12 ratings	21	−3	1	0.10	0.83	10	−3^a^	1	−0.10^a^	1.10	11	0	1	0.33	0.50
Baseline to end-point change in HoNOS-LD scale 12 ratings	17	−3	2	0.12	1.05	8	−3^a^	0	−0.50^a^	1.07	9	0	2	0.67	0.71
Baseline to mid-point change in HoNOS-LD total scores	21	−17	16	−1.62	6.15	10	−17^a^	2	−4.20^a^	6.27	11	−3^a^	16	0.73	5.26
Baseline to end-point change in HoNOS-LD total scores	17	−17	17	−0.41	6.63	8	−17^a^	1	−3.00^a^	5.90	9	−4^a^	17	1.89	6.70

HoNOS-LD, Health of the Nation Scales – Learning Disability.

a .Negative figures indicate a reduction in HoNOS-LD ratings and hence a clinical improvement.

Mauchly’s test assumption of sphericity violation on all the HoNOS-LD three-point testing done, and multivariate tests are reported. Specific test details and category is provided in Table 7.

**Table 7:** Mauchly’s test assumption of sphericity violation and multivariate tests on the HoNOS-LD

Investigated group	Assumption of sphericity *χ* ^2^ (2)	Mauchly’s test violated (yes/no)	Multivariate test (*ε*)	HoNOS-LD results over time	Inference
HoNOS-LD total scores (18 items), at the three time points	12.29, *P* = 0.002	Yes	0.641	*V* = 0.033, *F*(2,15) = 0.253, *P* = 0.780, *η* ^2^ = 0.010.	No significant change
HoNOS-LD total scores (18 items) for the subset of patients with constipation	12.91, *P* = 0.002	Yes	0.531	*V* = 0.229, *F*(2,6) = 0.891, *P* = 0.458, *η* ^2^ = 0.224	No significant change
HoNOS-LD total scores (18 items) for the subset of patients without constipation	2.90, *P* = 0.234	Yes	0.747	*V* = 0.083, *F*(2,7) = 0.317, *P* = 0.738, *η* ^2^ = 0.063	No significant change
HoNOS-LD Physical problems subscale (12 items) at the three time points	11.276, *P* = 0.004	Yes	0.654	*V* = 0.060, *F*(2,15) = 0.479, *P* = 0.628, *η* ^2^ = 0.014	No significant change
HoNOS-LD physical problems subscale (12 items) for the subset of patients with constipation at baseline	7.454, *P* = 0.024	Yes	0.584	*V* = 0.250, *F*(2,6) = 1.00, *P* = 0.422, *η* ^2^ = 0.162	No significant change
HoNOS-LD physical problems subscale (12 items) for the subset of patients without constipation at baseline	2.014, *P* = 0.365	No	0.800 (multivariate test done for consistency, although Mauchly’s test was not violated)	*V* = 0.500, *F*(2,7) = 3.500, *P* = 0.088, *η* ^2^ = 0.429	No significant change

HoNOS-LD, Health of the Nation Scales – Learning Disability

Focusing on the change in HoNOS-LD total scores, independent of severity, there was no significant difference between the constipated and non-constipated groups from baseline to mid-point (*U* = 33.00, *z* = −1.579, *P* = 0.119, *r* = 0.345), nor from baseline to end-point (*U* = 26.50, *z* = −0.920, *P* = 0.387, *r* = 0.223).

There was no significant difference in change in HoNOS-LD scale item 12 ratings, independent of severity, between the constipated and non-constipated groups between baseline and mid-point (*U* = 47.00, *z* = −0.714, *P* = 0.676, *r* = 0.156). There was a significant difference between the two groups in the change from baseline to end-point (*U* = 12.00, *z* = −2.606, *P* = 0.014, *r* = 0.632).

When considering total HoNOS-LD scores at the three time points, none of the three groups (all participants, participants with constipation at baseline, participants without constipation at baseline) reached statistical significance (*P* = 0.780, *P* = 0.458 and *P* = 0.387, respectively).

The two participants who had an increase in ACB scores at 3 months continued on the same higher score at 6 months. No further changes in ACB scores were noted over time.

## Discussion

We conducted a small feasibility study to explore the potential for using IBCPs in improving bowel functioning and preventing constipation in PwID in-patients on specialist intellectual disability units. We recruited 24 patients, which is a relatively low number, of which ten had constipation. This is consistent with most surveys of PwID, which found that around 33–50% of PwID had constipation.^8^ We demonstrated that measuring the outcome data in this group is feasible as we had robust data for 18 of the 24 patients at all three time points. Because of lack of funding, we were not able to measure the implementation of the IBCPs, which would strengthen future studies.

Most outcomes of our study did not show a difference before and after intervention. With such small numbers, type 2 errors are possible (not finding a difference when there is one). The one significant change was in the subscale of physical functioning (scale 12) of the HoNOS-LD between baseline and 6 months, where the change was better for those PwID who were constipated than those who were not. This one positive finding suggests some possibility that the intervention might lead to an improvement in the physical functioning of patients if study samples were larger.

The analysis results found no significant changes in outcomes from baseline to either the 3- or 6-month time point from the constipation questionnaire. However, it is noted that the patients generally had fairly good functioning at the baseline time point. For example, at the baseline time point, no patients had constipation requiring a general hospital admission, over 80% had a good fluid intake and over 80% had no assistance for their toilet routine. As a result of the good functioning at baseline, there may have been limited scope for improvement in outcomes during the course of the study. Further, there might be increased monitoring of physical health parameters during admission to a specialist intellectual disability unit, and daily availability of professionals able to diagnose, treat and introduce proactive measures to reduce constipation risk in patients.

There are well-known associations between psychotropic medication and antiseizure medication with constipation, and between drugs with high ACB and constipation.^28−31^ The data collected shows that a significant majority of participants had a high ACB. All but one were prescribed psychotropic medication, which is to be expected in an in-patient unit and in keeping with the need for admission in the first place. The cumulative anticholinergic effects are very likely to contribute to the overall physical health and bowel health of the cohort.

Although the analyses did not identify any significant changes over time in HoNOS-LD total scores, this could be because constipation was simply one of many clinical issues captured by the full 18 scales. The statistically significant difference in the level of change in the HoNOS-LD physical health question (item 12) (independent of severity) from baseline to end-point makes intuitive sense, as it is the variable that is most likely to be affected. It allows us to tentatively recommend further, larger-scale investigation in this area.

The process and results lead us to recommend that this feasibility study is suitable for further testing and longer-term implementation in the in-patient setting. We foresee that bowel charts could be successfully implemented into existing infrastructures on the units in the longer term, and sit comfortably alongside other daily health interventions (such as vital signs measurements, pre-existing bowel charts, physical activity schedules and diet plans). The IBCP would have the potential to amalgamate these health interventions into one holistic treatment plan, thus avoiding constraints on healthcare professional’s time or existing resources. The IBCPs have the potential for further personalisation in future projects and clinical practice. For example, additional free-text spaces could be included to support clear descriptions of the PwID’s comorbid conditions and prescribed medications to clearly highlight constipation-associated risks. Easy access to the plan by all healthcare professionals is important to ensure the plans are regularly utilised, and a suitable place for this may be as an appendix to the medication chart or in the purple folder once the person is discharged back into the community. To test the acceptability of the intervention, we propose that a survey could be disseminated among participants and healthcare professionals, to test the user-friendliness of the forms and their impact on daily care in the units. A larger and longer-term study would help clarify the utility of the IBCPs further and identify further needs for adaptation.

### Strengths and limitations

This study focuses on constipation, a common physical health condition affecting PwID, and one that is associated with considerable morbidity. It is also focused on PwID who are in-patients in specialist intellectual disability units, who represent a particularly unwell and vulnerable patient group. The questionnaire allowed us to gather standardised responses across the involved healthcare locations; however, this may have limited the depth of responses collected. Moreover, data related to the overall capacity, average length of stay and turnover of the units would have been desirable to inform interpretation of the data.

Phrases used in the questionnaire such as ‘assisted toilet routine’ were not defined and, in the absence of training or standardisation of rating approaches, this could have contributed to unreliability in outcome measures.

This study does not evaluate the changes first between baseline and 3 months and three to six months. It was felt that comparisons of baseline (i.e before any intervention started) and the two other data points would give the best representation of the impact and feasibility of the interventions overall. Such analysis could provide useful insights on the longer-term implications of the interventions in future studies.

In conclusion, this was a small study with all the limitations involved with a small sample. This study looks to focus on the process and implementation of the bowel plans and is not an attempt to show that they work. We could not measure whether the intervention of the IBCP was implemented by the whole ward team.

This is the first study we are aware of that has tried to measure the impact of IBCPs for PwID. We have shown that an interventional study for PwID that uses these outcome measures is feasible, and that there may be an impact on physical functioning. A clinical trial with adequate sample size may be indicated to measure the effectiveness of IBCPs.

## Supporting information

Gabrielsson et al. supplementary material 1 Gabrielsson et al. supplementary material

Gabrielsson et al. supplementary material 2 Gabrielsson et al. supplementary material

Gabrielsson et al. supplementary material 3 Gabrielsson et al. supplementary material

Gabrielsson et al. supplementary material 4 Gabrielsson et al. supplementary material

Gabrielsson et al. supplementary material 5 Gabrielsson et al. supplementary material

Gabrielsson et al. supplementary material 6 Gabrielsson et al. supplementary material

## Data Availability

Data used for the study can be obtained via the corresponding author, R.S., upon reasonable request.

## Supplementary material

The supplementary material is available online at https://doi.org/10.1192/bjo.2025.10814

## References

[1] American Psychiatric Association. Diagnostic and Statistical Manual of Mental Disorders (5th edn, text revision). APA, 2022.

[2] Alborz A , McNally R , Glendinning C. Access to health care for people with learning disabilities in the UK: mapping the issues and reviewing the evidence. J Health Serv Res Policy 2005; 10: 173–82. 16053595 10.1258/1355819054338997 PMC2020839

[3] Institute of Psychiatry, Psychology and Neuroscience. *Learning from Lives and Deaths – People with a Learning Disability and Autistic People (LeDeR)*. King’s College London, 2022 (https://www.kcl.ac.uk/ioppn/assets/fans-dept/leder-2022-takeaways.pdf).

[4] Liao P , Vajdic C , Trollor J , Reppermund S. Prevalence and incidence of physical health conditions in people with intellectual disability – a systematic review. PLoS One 2021; 16: e0256294. 34428249 10.1371/journal.pone.0256294 PMC8384165

[5] Tromans SJ , Teece L , Shankar R , Hassiotis A , Brugha T , McManus S. Primary care experiences of adults reporting learning disability: a probability sample survey. Br J Gen Pract 2024; 74: e845–53. 39374978 10.3399/BJGP.2024.0056 PMC11583036

[6] Brandt LJ , Prather CM , Quigley EM , Schiller LR , Schoenfeld P , Talley NJ. Systematic review on the management of chronic constipation in North America. Am J Gastroenterol 2005; 100(suppl 1): S5–21. 16008641 10.1111/j.1572-0241.2005.50613_2.x

[7] Kinnear D , Morrison J , Allan L , Henderson A , Smiley E , Cooper S-A. Prevalence of physical conditions and multimorbidity in a cohort of adults with intellectual disabilities with and without Down syndrome: cross-sectional study. BMJ Open 2018; 8: e018292. 10.1136/bmjopen-2017-018292 PMC5829598 29431619

[8] Robertson J , Baines S , Emerson E , Hatton C. Constipation management in people with intellectual disability: a systematic review. J Appl Res Intellect Disabil 2018; 31: 709–24. 29168259 10.1111/jar.12426

[9] Maslen C , Hodge R , Tie K , Laugharne R , Lamb K , Shankar R. Constipation in autistic people and people with learning disabilities. Br J Gen Pract 2022; 72: 348–51. 35772989 10.3399/bjgp22X720077 PMC9256070

[10] Glover G , Williams R , Oyinlola J. An observational cohort study of numbers and causes of preventable general hospital admissions in people with and without intellectual disabilities in England. J Intellect Disabil Res 2020; 64: 331–44. 32141168 10.1111/jir.12722

[11] NHS England. *Constipation Campaign Toolkit.* NHS England, 2023 (https://www.england.nhs.uk/long-read/constipation-campaign-toolkit/).

[12] NHS England. *Learning from Lives and Deaths – People with a Learning Disability and Autistic People (LeDeR) Policy, 2021.* NHS England, 2021 (https://www.england.nhs.uk/publication/learning-from-lives-and-deaths-people-with-a-learning-disability-and-autistic-people-leder-policy-2021/).

[13] NHS England and NHS Improvement. *LeDeR Disability Mortality Review (LeDeR) Programme: Action from Learning.*NHS England and NHS Improvement, 2019 (https://www.england.nhs.uk/wp-content/uploads/2019/05/action-from-learning.pdf).

[14] Laugharne R , Wilcock M , Rees J , Wainwright D , Newton N , Sterritt J , et al. Clinical characteristics of people with intellectual disability admitted to hospital with constipation: identifying possible specific high-risk factors. J Intellect Disabil Res 2024; 68: 277–84. 38031737 10.1111/jir.13108

[15] Bishop R , Laugharne R , Burrows L , Team CHAMPS , Ward S , Eustice S , et al. Laxative use in adults with intellectual disabilities: development of prescribing guidelines. BJPsych Open 2024; 10: e84. 38634310 10.1192/bjo.2024.50 PMC11060064

[16] Belsey J , Greenfield S , Candy D , Geraint M. Systematic review: impact of constipation on quality of life in adults and children. Aliment Pharmacol Ther 2010; 31: 938–49. 20180788 10.1111/j.1365-2036.2010.04273.x

[17] AlMutairi H , O’Dwyer M , Burke E , McCarron M , McCallion P , Henman MC. Laxative use among older adults with intellectual disability: a cross-sectional observational study. Int J Clin Pharm 2020; 42: 89–99. 31792735 10.1007/s11096-019-00942-z

[18] NHS England. *Poo Matters – Information for Families and Carers.* NHS England, 2019 (https://www.england.nhs.uk/wp-content/uploads/2019/05/constipation-resources-families-carers-stage-31-web.pdf).

[19] Abraham J , Purandare K , McCabe J , Wijeratne A , Eggleston E , Oak K , et al. An 8-year study of admissions and discharges to a specialist intellectual disability inpatient unit. J Appl Res Intellect Disabil 2022; 35: 569–76. 34931405 10.1111/jar.12967

[20] Bowen DJ , Kreuter M , Spring B , Cofta-Woerpel L , Linnan L , Weiner D , et al. How we design feasibility studies. Am J Prev Med 2009; 36: 452–7. 19362699 10.1016/j.amepre.2009.02.002 PMC2859314

[21] Ogrinc G , Davies L , Goodman D , Batalden PB , Davidoff F , Stevens D. SQUIRE 2.0 (Standards for QUality Improvement Reporting Excellence): revised publication guidelines from a detailed consensus process. BMJ Qual Saf 2016; 25: 986–92>. 10.1136/bmjqs-2015-004411 PMC5256233 26369893

[22] Krist AH , Tong ST , Aycock RA , Longo DR. Engaging patients in decision-making and behavior change to promote prevention. Stud Health Technol Inform 2017; 240: 284–302. 28972524 PMC6996004

[23] Laugharne R , Sawhney I , Perera B , Wainwright D , Bassett P , Caffrey B , et al. Chronic constipation in people with intellectual disabilities in the community: cross-sectional study. BJPsych Open 2024; 10: e55. 38425039 10.1192/bjo.2024.12 PMC10951845

[24] Roy A , Matthews H , Clifford P , Fowler V , Martin DM. Health of the Nation Outcome Scales for People with Learning Disabilities (HoNOS-LD). Br J Psychiatry 2002; 180: 61–6. 11772853 10.1192/bjp.180.1.61

[25] Painter J , Adams N , Ingham B , James M , Majid M , Roy A , et al. Review and update of the Health of the Nation Outcome Scales for People with Learning Disabilities (HoNOS-LD). Int J Soc Psychiatry 2023; 69: 1807–13. 37198876 10.1177/00207640231175773 PMC10657504

[26] Painter J, Purandare K, McCabe J, Roy A, Shankar R. Investigating the component structure of the Health of the Nation Outcomes Scales for people with Learning Disabilities (HoNOS-LD). *Int J Social Psychiatry* [Epub ahead of print] 12 Mar 2025. Available from: 10.1177/00207640251323819. PMC12357968 40071659

[27] King R , Rabino S. ACB Calculator, 2023 (https://www.acbcalc.com/).

[28] Xu Y , Amdanee N , Zhang X. Antipsychotic-induced constipation: a review of the pathogenesis, clinical diagnosis, and treatment. CNS Drugs 2021; 35: 1265–74. 34427901 10.1007/s40263-021-00859-0

[29] Coleman J , Spurling G. Constipation in people with learning disability. BMJ 2010; 340: c222. 20103526 10.1136/bmj.c222

[30] Kelly K , Posternak M , Alpert JE. Toward achieving optimal response: understanding and managing antidepressant side effects. Dialogues Clin Neurosci 2008; 10: 409–18. 19170398 10.31887/DCNS.2008.10.4/kkelly PMC3181894

[31] Tharian R, Hicks M, Sahadevan S, Patteril E, Chester V, Alexander R, et al. Anticholinergic burden in people with intellectual disability in psychiatric inpatient units: practice and audit recommendations. *J Mental Health Res Intell Disabil* [Epub ahead of print] 23 May 2025. Available from: 10.1080/19315864.2025.2507640.

