# Supplementary material for: Using individualised bowel care plans to improve clinical outcomes in specialist intellectual disability mental health units in England and Wales: quality improvement project
Source: BJPsych Open. 2025 Aug 18;11(5):e186. doi: 10.1192/bjo.2025.10814 (PMC12451552; doi:10.1192/bjo.2025.10814)
Supplement: Gabrielsson et al. supplementary material 2 — Gabrielsson et al. supplementary material [file S2056472425108144sup002.docx]

**Constipation Questionnaire**

| **Name** |  |
| --- | --- |
| **Age** |  |
| **Gender** |  |
| **Person providing information** |  |

| Residence in last month | Hospital ☐  Nursing Home ☐  LD Residential Home ☐  Supported Living ☐  Domiciliary Care ☐  Community – with family ☐  Community – independently ☐ |
| --- | --- |
| Degree of ID | Mild ☐  Moderate ☐  Severe-profound ☐ |
| Down syndrome | Yes ☐ No ☐ |
| Any other known genetic syndrome: |  |
| Cerebral palsy | Yes ☐ No ☐ |
| Epilepsy | Yes ☐ No ☐ |
| Diagnosed mental illness | None ☐ Psychosis ☐ Non-psychotic ☐ |
| Dysphagia | Yes ☐ No ☐ |
| Obesity | Yes ☐ No ☐ |
| Diabetes | Yes ☐ No ☐ |
| Autism | Yes ☐ No ☐ |

**Constipation**

| Number of bowel movement per week (last month): | More than two ☐  Two or fewer ☐  Don’t know ☐ |
| --- | --- |
| Incontinence of faeces | Daily ☐  Less than daily ☐  Never ☐  Don’t know ☐ |
| Laxative use (last month) | Never ☐  Less than three times weekly ☐  Three or more times weekly ☐  Prescribed, don’t know usage ☐ |
| Impact of constipation: |  |
| Admitted to hospital with constipation | Yes ☐  No ☐  Don’t know ☐ |
| Required surgery following admission with constipation | Yes ☐  No ☐  Don’t know ☐ |

**Risk factors**

| **Record all medications taken on a regular basis, (e.g. daily or weekly).**  *Please include prescription and non-prescription medications, over-the-counter medications, vitamins, and herbal and alternative medications.* |
| --- |

| **Diet** | Had advice on diet and implemented ☐  Had advice on diet but not implemented ☐  No advice on diet received but diet maintained ☐  No diet and little focus ☐  Liquidised diet only ☐  Tube feeding ☐  If advice received, who gave this? |
| --- | --- |
| **Fluid intake** | Fluid intake difficult ☐  Fluid intake good; 6-8 drinks daily ☐  Don’t know ☐  If difficult what is the average daily intake in ml over last month? |
| **Toileting:** | **Level of independence**  Independently toilets ☐  Requires support with toileting ☐  Never uses toilet as incontinent ☐  Not sure ☐  **Routine**  No routine ☐  Assisted routine ☐  Don’t know ☐  **Seat**  Normal toilet seat ☐  Raised toilet seat ☐  Use of foot stool or aids ☐  Not relevant as incontinent ☐  Not sure ☐ |
| **Mobility:** | Good with exercise ☐  Good without much exercise ☐  Impaired ☐  Largely immobile ☐  Unable to get out of bed ☐ |

Laugharne, R., Sawney, I., Perera, B., Wainwright, D., Bassett, P., Caffrey, B., O’Dwyer, M., Lamb., Wilcock, M., Roy, A., Oak, K., Eustice, S., Newton, N., Sterritt, J., Bishop, R., & Shankar, R. (2024). Chronic constipation in people with intellectual disabilities in the community: a cross sectional study. *BJPsych Open*

For electronic copies of this questionnaire please email cft.cider@nhs.net.
