# Supplementary material for: Using individualised bowel care plans to improve clinical outcomes in specialist intellectual disability mental health units in England and Wales: quality improvement project
Source: BJPsych Open. 2025 Aug 18;11(5):e186. doi: 10.1192/bjo.2025.10814 (PMC12451552; doi:10.1192/bjo.2025.10814)
Supplement: Gabrielsson et al. supplementary material 3 — Gabrielsson et al. supplementary material [file S2056472425108144sup003.pdf]

Poo matters

Information for families and carers

Some people with a learning disability are more at risk of constipation than the general population. Reviews into the deaths of people with a learning disability have shown us that too many people are dying from constipation.

We have developed this leaflet to help families and carers of people with a learning disability know the signs of constipation and what to do if you think someone is constipated.

Constipation means having trouble pooing

Top tips for a healthy poo

- Healthy diet – eat lots of things which are high in fibre like fruit and vegetables, wholegrain cereals and wholemeal bread.
- Drink six to eight glasses of liquid a day.
- Stay active and exercise.

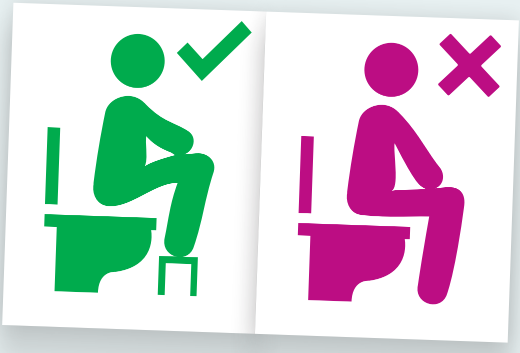

Top toilet tips

- Relax when sat on the toilet.
- Putting feet on a low stool and leaning forward can help.

Medication

- Ask your health professional if there are any prescribed medications that the person is taking which might cause constipation or make it worse.
- Don't stop laxatives or any other medication without talking to a health professional.

Asking for help

It can be difficult to tell a health professional why you are worried, so we've developed this guide with examples to help you.

How to ask for help

Tell the person you are talking to:

- your name and your relationship to the person you are calling about.
- the name of the person you are calling about.
- what is worrying you about the person.

I am Sue, John's mother. I am calling about John Smith because he hasn't had a poo for five days and now when he does go to the toilet his poo is very runny.

Tell the person why you are worried

What has changed about the person you care for?

John is 42 years old and has a learning disability. John has had constipation a few times before and has ended up in hospital with it but he doesn't take medication for it at the moment. I'm worried because he is really withdrawn and that's not like him. I know how important it is to make sure he isn't constipated again because I've heard about people with a learning disability dying from constipation.

What help do you need?

Tell the person you are talking to what help you think you need. That might be advice on whether to give the person laxatives and how much to give them or it might be something different.

I think John is constipated and I need someone to help me with the medication and how much I should give him.

Or you might feel that a health professional needs to see them and you need help to arrange that quickly.

I want someone to see John today so he can get the right treatment and won't need to go into hospital.

Warning signs of constipation

Changes in behaviour – is the person you care for doing things they wouldn't normally do?

Having a poo less often than normal

Poo seems hard and the person is finding it difficult to pass

Tummy ache, not eating or feeling sick

Swollen or bloated tummy

Poo in underwear

Signs of bleeding when trying to poo

Care plan

To help the person you care for have a healthy poo routine you can photocopy this sheet as many times as you want and use it as a prompt. You can show it to a health professional if you are worried about anything.

Name: .... Date this advice agreed: ..../.........../...............

I have a bowel condition which can cause severe constipation and this advice is vital to keep me well:

....

My condition can be kept under control by:

1 Eating well

I need to eat a high fibre diet – things like fruit, vegetables, wholegrain cereals and wholemeal bread.

I need to avoid foods that constipate me such as ....

(eg meat, refined foods)

2 Drink plenty

I need to drink .... glasses of water or other fluids every day.

3 Exercise

I need to exercise every day.

I like to ....

....

....

(give an example such as walking, swimming etc)

4 Going to the toilet

I need reminding regularly to go to the toilet.

It is healthy to have a poo every day which is soft and I can do easily, so remind me every day.

I usually have a poo .... times a day / every .... (days)

I might find it easier to poo if I put my feet on a low foot stool when I sit on the toilet.

I know it is important that I have privacy, but it is more important that someone keeps a record about my poo so that I can stay well.

5 Medication

Please make sure I take the laxatives the doctor prescribes for me as instructed (if this is the case).

If I have a large loose poo, especially after I haven't had a poo for three or more days, this is probably backlog and not diarrhoea. Please do not stop my medication as I will get constipated again very quickly.

6 Warning signs

If I haven't had a poo for three or more days then you need to

....

(describe what needs to happen eg take extra laxative or talk to a health professional)

When I am constipated you might notice these things:

- Hard/large, small poos, tummy ache and bloating.
- I pass lots of smelly wind or I may stop passing wind - that is a sign of severe constipation.
- Poo on my underwear (this might be overflow incontinence).
- I might not behave in the same way I do normally and I might not be able to tell you what is the matter with me.

(please add anything specific to the person) ....

Where can I find more information about constipation in people with a learning disability?

Dimensions have produced a simple video called 'Poo and you' which talks about how dangerous constipation can be for people with a learning disability. You can find the video at this link youtu.be/1TlcefPXjgg

It is important not to confuse loose poo with diarrhoea as it may be constipation – if you are worried talk to a health professional.

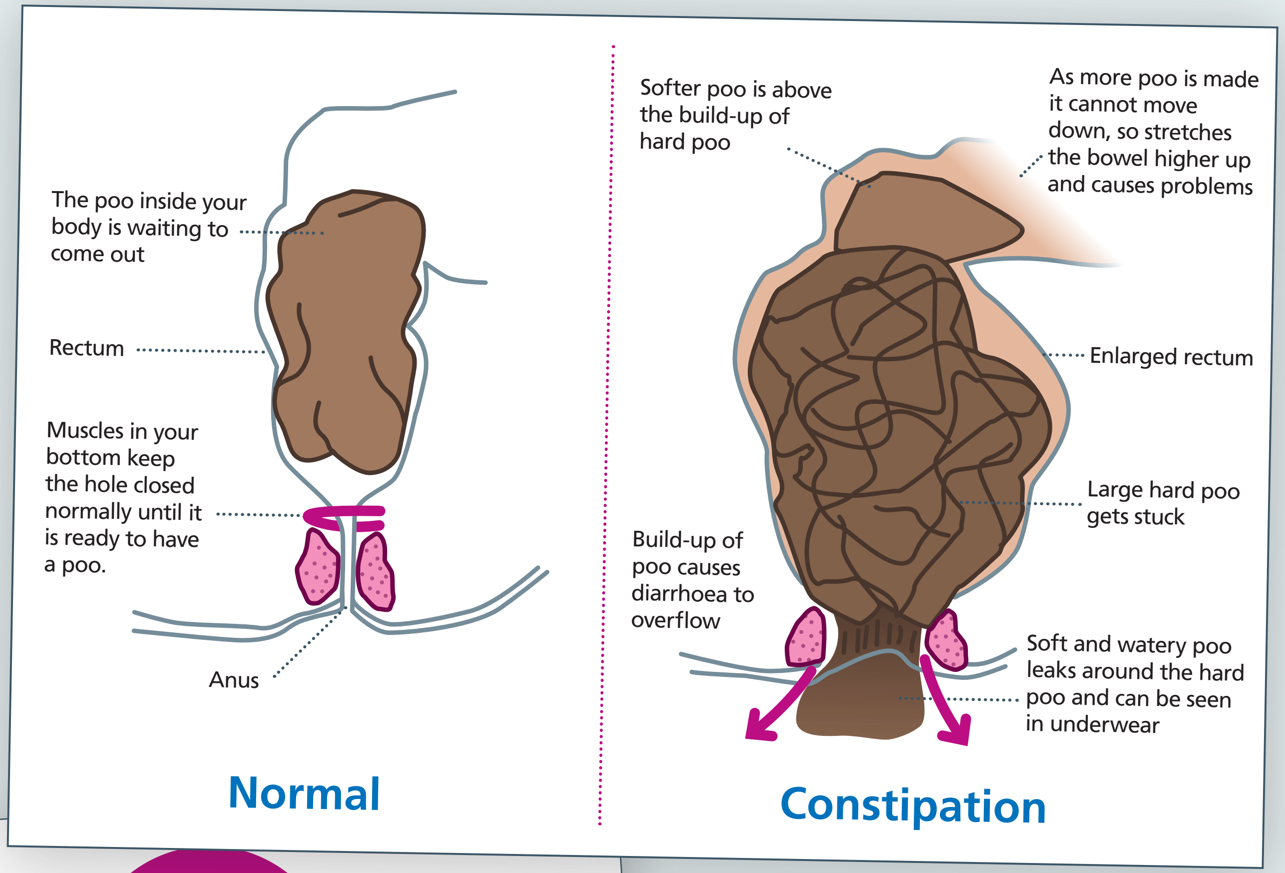

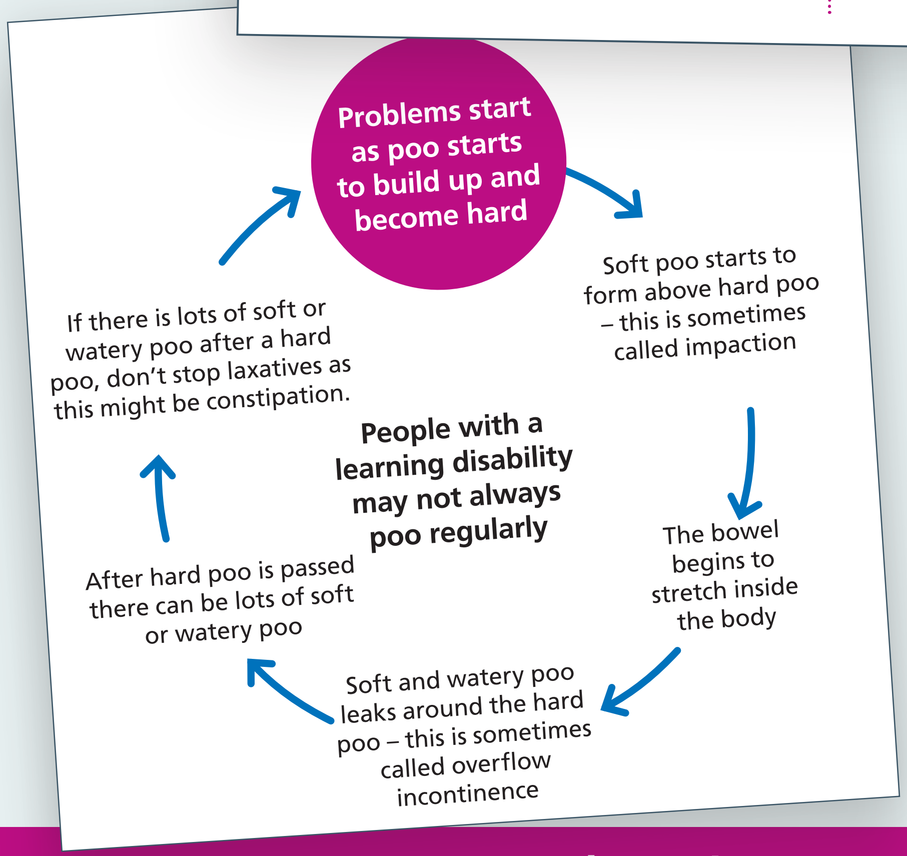

#TakeActionSaveLives
