# Supplementary material for: Using individualised bowel care plans to improve clinical outcomes in specialist intellectual disability mental health units in England and Wales: quality improvement project
Source: BJPsych Open. 2025 Aug 18;11(5):e186. doi: 10.1192/bjo.2025.10814 (PMC12451552; doi:10.1192/bjo.2025.10814)
Supplement: Gabrielsson et al. supplementary material 4 — Gabrielsson et al. supplementary material [file S2056472425108144sup004.docx]

**Supplementary information 6: Prescribed medication list of each participant and associated ACB scores**

| **Participant** | **Medication at baseline** | **ACB score (BL)** | **Medication at 3 months** | **ABC score  (3 months)** | **Medication at 6 months** | **ACB score at 6 months** |
| --- | --- | --- | --- | --- | --- | --- |
| **1** | Atomoxetine  Epilim Chrono Lorazepam PRN Colecalciferol | 2 | Atomoxetine  Epilim Chrono  Lorazepam PRN Colecalciferol | 2 | Lost to follow up | N/A |
| **2** | Zuclopenthixol dihydrochloride Depakote Procyclidine Amisulpride Medroxyprogesterone  Metformin MR Allopurinol Colecalciferol  Lorazepam PRN Promethazine PRN | 10 | Zuclopenthixol dihydrochloride Depakote Procyclidine Amisulpride Medroxyprogesterone  Metformin MR Movicol Colecalciferol Lorazepam PRN Promethazine PRN | 10 | Zuclopenthixol dihydrochloride Depakote Procyclidine Amisulpride Medroxyprogesterone  Metformin MR Movicol Colecalciferol Lorazepam PRN Promethazine PRN | 10 |
| **3** | Olanzapine  Pregabalin  Atomoxetine Colecalciferol Lorazepam PRN Promethazine PRN Paracetamol PRN | 7 | Sertraline Aripiprazole Olanzapine Pregabalin Atomoxetine Colecalciferol Movicol Lorazepam PRN Promethazine PRN Paracetamol PRN | 9 | Sertraline Aripiprazole Olanzapine Pregabalin Atomoxetine Colecalciferol Movicol Lorazepam PRN Promethazine PRN Paracetamol PRN | 9 |
| **4** | Olanzapine  Sertraline  Clonazepam  Lorazepam PRN Paracetamol PRN Trimethoprim Omeprazole Colecalciferol | 7 | Olanzapine Sertraline  Pregabalin Clonazepam Lorazepam PRN Paracetamol PRN Timethoprim Omeprazole Colecalciferol | 7 | Olanzapine Sertraline Clonazepam Lorazepam PRN Paracetamol PRN Nitrofurantoin Omeprazole Colecalciferol Ferrous Fumarate | 7 |
| **5** | Quetiapine Sertraline Zopiclone Lorazepam PRN Promethazine PRN Atorvastatin Colecalciferol Lansoprazole  Propranolol Gaviscon PRN Laxido  Senna PRN Paracetamol PRN | 9 | Quetiapine Sertraline Zopiclone Lorazepam PRN Promethazine PRN Atorvastatin Colecalciferol Lansoprazole  Propranolol Gaviscon PRN Laxido  Senna PRN Paracetamol PRN Sudocrem PRN | 9 | - | - |
| **6** | Aripiprazole Semisodium Valproate Lorazepam PRN Promethazine PRN Zopiclone Colecalciferol  Loratidine  Senna Fusidic acid Paracetamol PRN  Hydromol cream PRN | 7 | Aripiprazole Semisodium Valproate Lorazepam PRN Promethazine PRN Zopiclone Colecalciferol  Loratidine  Senna Fusidic acid Paracetamol PRN  Hydromol cream PRN | 7 | Aripiprazole Semisodium Valproate Lorazepam PRN Promethazine PRN Zopiclone Colecalciferol  Loratidine  Senna Fusidic acid Paracetamol PRN  Hydromol cream PRN | 7 |
| **7** | Quetiapine Risperidone Priadel Levetiracetam Circadin Procyclidine Lorazepam PRN Colecalciferol Fexofenadine  Lactulose Omeprazole  Paracetamol PRN Eumovate PRN | 9 | Quetiapine Risperidone Priadel Levetiracetam Circadin Procyclidine Lorazepam PRN Colecalciferol Fexofenadine  Lactulose Omeprazole  Peptac Laxido PRN Paracetamol PRN Eumovate PRN | 9 | Quetiapine Risperidone Priadel Levetiracetam Circadin Procyclidine Lorazepam PRN Colecalciferol Fexofenadine  Lactulose Omeprazole  Paracetamol PRN Eumovate PRN | 9 |
| **8** | Fluoxetine  Methylphenidate XL Pregabalin Zopiclone Lorazepam PRN Promethazine PRN Omeprazole  Desogestrel  Paracetamol | 6 | Fluoxetine  Methylphenidate XL Pregabalin Atomoxetine Zopiclone Lorazepam PRN Promethazine PRN Omeprazole Desogestrel | 6 | - |  |
| **9** | Olanzapine Haloperidol Clonazepam  Depakote Lorazepam Promethazine Zopiclone PRN Procyclidine PRN Buspirone Hyoscine hydrobromide Beclomethasone Atorvastatin  Docusate  Colecalciferol Codeine PRN  Paracetamol PRN  Ibuprofen PRN Lactulose PRN | 13 | Olanzapine  Clonazepam  Depakote  Buspirone  Hyoscine hydrobromide Atorvastatin  Sodium Docusate  Colecalciferol  Desmopressin  Omeprazole  Nitrofurantoin Laxido PRN | 8 | - | - |
| **10** | Sodium Valproate  Levetiracetam Melatonin Memantine Lorazepam PRN Buccal Midazolam PRN Levothyroxine Acetylcysteine Laxido Fostair inhaler Peptac  Omeprazole  Loratadine  Paracetamol PRN | 5 | Sodium Valproate Levetiracetam Melatonin Memantine Lorazepam PRN Buccal Midazolam PRN Levothyroxine  Nacsys  Laxido PRN Fostair inh  Peptac  Omeprazole  Loratadine  Paracetamol PRN Hydromol  Med-derma S barrier cream PRN Salbutamol inh PRN  Hylo night eye ointment  Sodium fluoride paste | 5 | Sodium Valproate Levetiracetam Melatonin Memantine Lorazepam PRN Buccal Midazolam PRN Levothyroxine  Nacsys  Laxido PRN Fostair inh  Peptac  Omeprazole  Loratadine  Paracetamol PRN Hydromol  Med-derma S barrier cream PRN Salbutamol inh PRN  Hylo night eye ointment  Sodium fluoride paste | 5 |
| **11** | Carbamazapine Trihexyphenidyl Mirtazapine Risperidone Melatonin Temazepam Brivaracetam Lorazepam PRN Lactulose Fortisip Omeprazole  Multivitamins  Cetirizine  Desmopressin  Accrete D3  Senna PRN Nutricrem dessert  Duraphat toothpaste Paracetamol PRN Barrier cream PRN | 8 | Carbamazapine Trihexyphenidyl Mirtazapine Risperidone Melatonin Temazepam Brivaracetam Lorazepam PRN Lactulose Fortisip Omeprazole  Multivitamins  Cetirizine  Desmopressin  Accrete D3  Senna PRN Nutricrem dessert  Duraphat toothpaste Paracetamol PRN Zerolon cream PRN | 8 | Carbamazapine Trihexyphenidyl Mirtazapine Risperidone Melatonin Temazepam Brivaracetam Lorazepam PRN Lactulose Fortisip Omeprazole  Multivitamins  Cetirizine  Desmopressin  Accrete D3  Senna PRN Nutricrem dessert  Duraphat toothpaste Paracetamol PRN Barrier cream PRN | 8 |
| **12** | Sodium Valproate Hyoscine Hydrobromide Cetirizine Lansoprazole Fresubin supplement  Laxido PRN Paracetamol PRN  Ibuprofen gel PRN | 6 | Lost to follow up - deceased | N/A | N/A | N/A |
| **13** | Phenytoin  Brivaracetam  Glycopurronium bromide Gabapentin Midazolam PRN Sodium valproate  Clonazepam  Atorvastatin  Lactulose  Nutrilis thickener Paracetamol PRN Buccal  Laxido | 6 | Phenytoin  Brivaracetam  Glycopurronium bromide Gabapentin Midazolam PRN Sodium valproate  Clonazepam  Atorvastatin  Lactulose  Nutrilis thickener Paracetamol PRN Buccal  Laxido Balneum plus | 6 | Phenytoin  Brivaracetam  Glycopurronium bromide Gabapentin Midazolam PRN Sodium valproate  Clonazepam  Atorvastatin  Lactulose  Nutrilis thickener Paracetamol PRN Buccal  Laxido | 6 |
| **14** | Carbamazpine Mirtazapine Quetiapine Loratadine Ventolin inh  Clenil modulate inh Mometasone nasal spray  Clopidogrel  Omeprazole Senna PRN  Paracetamol PRN Movicol PRN | 6 | Carbamazpine Mirtazapine Quetiapine Loratadine Ventolin inh  Clenil modulate inh Mometasone nasal spray  Clopidogrel  Omeprazole Fenbid  Drapolene PRN  Hydromol  Sodium fluoride toothpaste  Glycerol suppository PRN- never used Senna PRN  Paracetamol PRN Movicol PRN | 6 | Carbamazpine Mirtazapine Quetiapine Loratadine Ventolin inh  Clenil modulate inh Mometasone nasal spray  Clopidogrel  Omeprazole Fenbid  Drapolene PRN  Hydromol  Sodium fluoride toothpaste  Glycerol suppository PRN- never used Senna PRN  Paracetamol PRN Movicol PRN | 6 |
| **15** | Haloperidol  Sertraline  Semaglutide  Metformin  Atorvastatin  Amlodipine  Omeprazole  Promethazine PRN  Lorazepam PRN Paracetamol PRN Olive oil ear drops Ellipta inh Mometasone spray  Hydromol cream | 8 | Haloperidol  Sertraline  Semaglutide  Metformin  Atorvastatin  Amlodipine  Omeprazole  Promethazine PRN  Lorazepam PRN  Ellipta inh  Mometasone spray Hydromellose drops | 8 | Haloperidol  Sertraline  Semaglutide  Metformin  Atorvastatin  Amlodipine  Omeprazole  Promethazine PRN  Lorazepam PRN  Ellipta inh  Mometasone spray Hydromellose drops | 8 |
| **16** | Venlafaxine Aripiprazole Diazepam Melatonin Hyoscine butylbromide  Amitriptyline Flucloxacillin Famotidine  Nicotine patch Laxido  Dalteparin  Montelukast  Fexofenadine  Flutiform inh  Naproxen  Alverine  Furosemide  Docusate sodium  Desogestrel  Laxido T PRN Paracetamol PRN  Salbutamol PRN  Codeine PRN  Oramorph PRN  Lorazepam PRN | 12 | Venlafaxine Aripiprazole Diazepam Melatonin Hyoscine butylbromide  Amitriptyline Famotidine  Nicotine patch Laxido  Dalteparin  Montelukast  Fexofenadine  Flutiform inh  Naproxen  Cetirizine Chlorphenamine Alverine  Furosemide  Docusate sodium  Desogestrel  Parrafin  Flamizine Laxido T PRN Paracetamol PRN  Salbutamol PRN  Oxycodone PRN  Promethazine PRN | 15 | Venlafaxine Aripiprazole Diazepam Melatonin Amitriptyline Nicotine chewing gum Dalteparin  Montelukast  Fexofenadine  Flutiform inh  Alverine  Furosemide  Penicillin Dosusate sodium  Desogestrel  Parrafin  Cetirizine Chlorphenamine Flamizine  Promethazine PRN Paracetamol PRN Salbutamol inh PRN Oxycodone PRN | 12 |
| **17** | Colecalciferol Desogestrel Laxido | 0 | Colecalciferol Desogestrel Laxido | 0 | Colecalciferol Desogestrel Laxido | 0 |
| **18** | Sertraline Aripiprazole Colecalciferol | 2 | Sertraline Aripiprazole Colecalciferol | 2 | Sertraline  Aripiprazole  Colecalciferol | 2 |
| **19** | Sodium Valproate Risperidone  Colecalciferol  Loratadine | 3 | Sodium Valproate  Risperidone  Colecalciferol  Loratadine | 3 | Sodium Valproate  Risperidone  Colecalciferol  Loratadine | 3 |
| **20** | Sodium Valproate  Donepezil Memantine  Loratadine  Lansoprazole  Paracetamol | 3 | Sodium Valproate  Donepezil Memantine  Loratadine  Lansoprazole  Paracetamol | 3 | Sodium Valproate  Donepezil Memantine  Loratadine  Lansoprazole  Paracetamol | 3 |
| **21** | Aripiprazole  Sertraline Clonazepam Codeine  Colecalciferol | 3 | Aripiprazole  Sertraline Clonazepam Codeine  Colecalciferol | 3 | Aripiprazole  Sertraline Clonazepam Codeine  Colecalciferol | 3 |
| **22** | - |  | - |  | - |  |
| **23** | - |  | - |  | - |  |
| **24** | - |  | - |  | - |  |
