# Supplementary material for: Using individualised bowel care plans to improve clinical outcomes in specialist intellectual disability mental health units in England and Wales: quality improvement project
Source: BJPsych Open. 2025 Aug 18;11(5):e186. doi: 10.1192/bjo.2025.10814 (PMC12451552; doi:10.1192/bjo.2025.10814)
Supplement: Gabrielsson et al. supplementary material 6 — Gabrielsson et al. supplementary material [file S2056472425108144sup006.pdf]

NHS
Cheshire and Wirral Partnership
NHS Foundation Trust

R&D Office
Countess of Chester Health Park
Liverpool Road
CHESTER
CH2 1BQ

Tel: 0151 488 7326

22nd November, 2023

Drs Rohit Shankar and Richard Laugharne
University of Plymouth/Cornwall Partnership NHS Foundation Trust

Ref: KB/07/23

Dear Rohit and Richard,

Re: Service Evaluation Decision Letter

Project Title: Constipation in people with intellectual disability (PwID) on inpatient units

Further to your request for governance approval, we are pleased to inform you that Cheshire and Wirral Partnership NHS Foundation Trust has approved your service evaluation. Please confirm receipt of this letter to activate approval.

Trust approval covers carrying out the studies as specified in the documents submitted:

- Project protocol, v1, 01/11/2023
- Information sheet, v1, 02/11/2023
- Consent/Consultee form, v1, 01/11/2023

Your responsibilities:

Your responsibilities when conducting service evaluations are to accord with governance procedures and your study may be monitored by the Research Office. Should you wish to publish your results, approval must be sought to protect the Trust's corporate identity and any information going into the public domain needs to be screened.

Please inform the Research Office once you have finished your study and submit a final summary report so that we may share findings for the interest and potential benefit of staff, service users and carers.

Please contact the Research Office should you require any further information. You may need this letter as proof of your approval.

May I wish you every success with your study.

Yours sincerely

Phil Elliott

Dr Phil Elliott
Senior Research Facilitator

© Cheshire and Wirral Partnership NHS Foundation Trust
